# Phenotypic patient profiling for improved implementation of guideline-directed medical therapy: An exploratory analysis in a large real-world chronic heart failure cohort

**DOI:** 10.3389/fphar.2023.1081579

**Published:** 2023-03-09

**Authors:** Sumant P. Radhoe, Pascal R. D. Clephas, Gerard C. M. Linssen, Remko M. Oortman, Frank J. Smeele, Annemarie A. Van Drimmelen, Henk-Jan Schaafsma, Paul H. Westendorp, Hans-Peter Brunner-La Rocca, Jasper J. Brugts

**Affiliations:** ^1^ Department of Cardiology, Thorax Center, Erasmus MC, University Medical Center Rotterdam, Rotterdam, Netherlands; ^2^ Department of Cardiology, Hospital Group Twente, Hengelo, Netherlands; ^3^ Department of Cardiology, Bravis Hospital, Bergen op Zoom, Netherlands; ^4^ Department of Cardiology, Slingeland Hospital, Doetinchem, Netherlands; ^5^ Department of Cardiology, Amphia Hospital, Breda, Netherlands; ^6^ Department of Cardiology, Hospital Gelderse Vallei, Ede, Netherlands; ^7^ Department of Cardiology, Rivas Beatrix Hospital, Gorinchem, Netherlands; ^8^ Department of Cardiology, Maastricht University Medical Center, Maastricht, Netherlands

**Keywords:** heart failure, guideline-directed medical therapy, clinical profiles, guideline implementation, pharmacotherapy, phenotype, personalized medicine

## Abstract

**Aims:** Implementation of guideline-recommended pharmacological treatment in heart failure (HF) patients remains challenging. In 2021, the European Heart Failure Association (HFA) published a consensus document in which patient profiles were created based on readily available patient characteristics and suggested that treatment adjusted to patient profile may result in better individualized treatment and improved guideline adherence. This study aimed to assess the distribution of these patient profiles and their treatment in a large real-world chronic HF cohort.

**Methods and results:** The HFA combined categories of heart rate, blood pressure, presence of atrial fibrillation, chronic kidney disease, and hyperkalemia into eleven phenotypic patient profiles. A total of 4,455 patients with chronic HF and a left ventricular ejection fraction ≤40% with complete information on all characteristics were distributed over these profiles. In total, 1,640 patients (36.8%) could be classified into one of the HFA profiles. Three of these each comprised >5% of the population and consisted of patients with a heart rate >60 beats per minute with normal blood pressure (>90/60 mmHg) and no hyperkalemia.

**Conclusion:** Nearly forty percent of a real-world chronic HF population could be distributed over the eleven patient profiles as suggested by the HFA. Phenotype-specific treatment recommendations are clinically relevant and important to further improve guideline implementation.

## Introduction

Heart failure (HF) is often characterized as a global pandemic, with recent reports estimating the number of HF patients at 64.3 million. (Ambrosy et al., 2014; Global and regional, 2018; Groenewegen et al., 2020). HF is among the leading causes of mortality and morbidity and causes high healthcare-related costs, especially due to the high (re)hospitalization rates. (Ambrosy et al., 2014; van Riet et al., 2016; Groenewegen et al., 2020; Urbich et al., 2020). Despite improvements in survival of HF patients over the last decade, which can partly be attributed to advances made in HF drug therapy, mortality remains high with survival rates of 56.7% and 34.9% for 5 and 10 years, respectively (Jones et al., 2019). Left ventricular ejection fraction (LVEF) is used as a phenotypic marker to categorize HF into separate entities with different underlying pathophysiological mechanisms, namely, HF with a reduced, mildly reduced, and preserved ejection fraction (HFrEF, HFmrEF, and HFpEF, respectively). (Borlaug and Redfield, 2011; Paulus and Tschope, 2013; Groenewegen et al., 2020). The current European Society of Cardiology (ESC) HF Guidelines contain four class I drug recommendations for the treatment of HFrEF patients as a result of numerous RCTs that have been conducted over the past years. (McDonagh et al., 2021). These are angiotensin receptor-neprilysin inhibitor (ARNI)/angiotensin-converting enzyme inhibitor (ACEi)/angiotensin receptor blocker (ARB), betablocker, mineralocorticoid receptor antagonist (MRA), and sodium-glucose co-transporter two inhibitor (SGLT2i). While the ESC HF Guidelines make it very clear that all four drug classes should be used in HFrEF therapy, it is still challenging to implement this into daily clinical practice. (McDonagh et al., 2021; Seferovic et al., 2021). Several HF registry studies have indeed shown room for improvement in guideline adherence. (Crespo-Leiro et al., 2016; Komajda et al., 2017; Greene et al., 2018; Brunner-La Rocca et al., 2019; Savarese et al., 2021; Savarese et al., 2023). Patients are frequently treated with regimens that do not include all cornerstone HF drugs, and with doses lower than those recommended in the guidelines. Lately, there has been attention for a rapid sequencing strategy for implementation of HF therapy as opposed to the conventional approach of up-titrating a drug class before adding a new one, and is supported by data from several studies. (Savarese et al., 2021; Shen et al., 2022; D'Amario et al., 2022). Multiple viewpoints have been published to give shape to the rapid sequencing strategy and to provide guidance to clinicians. (Greene et al., 2021; McMurray and Packer, 2021; Packer and McMurray, 2021; Beldhuis et al., 2022). One of these viewpoints entails selecting the initial therapy and sequencing strategy based on the clinical parameters of the patient, resulting in different patient “profiles”. (Rosano et al., 2021; McMurray and Docherty, 2022). Heart rate, low blood pressure, impaired renal function, and serum potassium are indeed commonly encountered factors that interfere with the initiation and up-titration of HF drugs, with the practical guidance supplement of the ESC HF Guidelines often advising to down-titrate or even discontinue certain drug classes based on these parameters. (McDonagh et al., 2021). In 2021, the Heart Failure Association (HFA) of the ESC published a position paper in an effort to offer all patients a regimen as close to guideline-directed medical therapy (GDMT) as possible by advocating for personalized drug treatment instead of a ‘one size fits all’ approach. (Rosano et al., 2021). In order to do so, the HFA postulated several patient profiles based upon clinical patient characteristics that can be used for specific treatment recommendations and may be relevant in the process of drug implementation and up titration. (Rosano et al., 2021). However, the prevalence of these profiles and associated patient characteristics and treatment variations in the HFrEF population in clinical practice are not yet known.

## Aims

This study aimed to assess the distribution and treatment characteristics of the patient profiles as previously proposed by the HFA in a large real-world chronic HF cohort.

## Materials and methods

Data from the CHECK-HF registry were used to identify the patient profiles. The design and methods of the registry have been published previously. (Brugts et al., 2018; Brunner-La Rocca et al., 2019; Radhoe et al., 2022). In short, the CHECK-HF registry enrolled patients with chronic heart failure in an outpatient setting. The study was conducted in accordance with the declaration of Helsinki and ethical approval was provided by the medical ethical committee of the Maastricht University Medical Center, the Netherlands.

For this analysis, patients with a left ventricular ejection fraction (LVEF) ≤40% were selected, leaving a total of 6,256 patients. Next, only patients with available information on heart rate, systolic and diastolic blood pressure, serum potassium, estimated glomerular filtration rate and atrial fibrillation were analyzed and distributed over the HFA profiles (N = 4,455).

**FIGURE 1:**
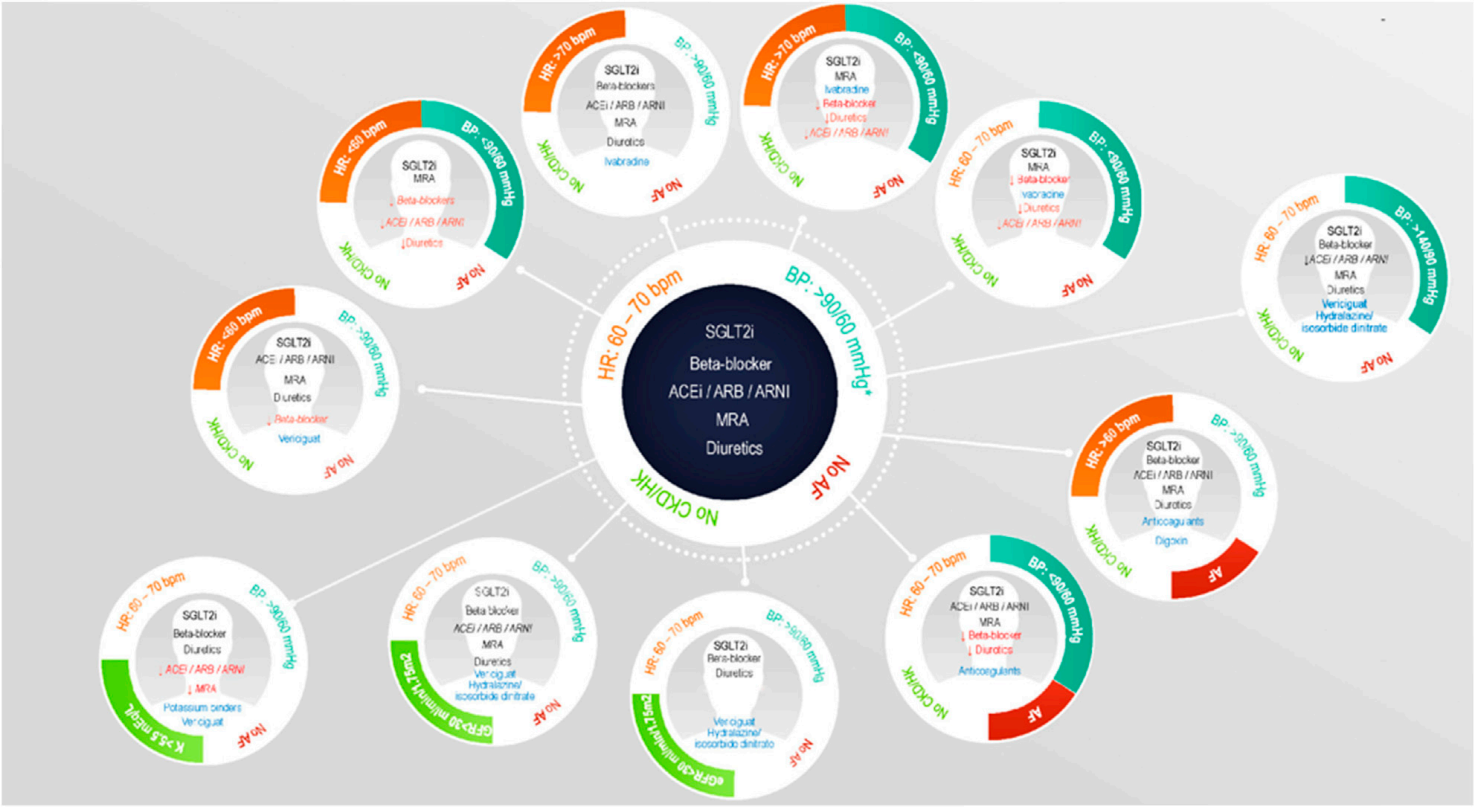
Overview of the eleven patient profiles as proposed by the Heart Failure Association. Reprinted from European Journal of Heart Failure, Volume 23, Issue 6, Rosano et al., Patient profiling in heart failure for tailoring medical therapy. A consensus document of the Heart Failure Association of the European Society of Cardiology, Pages 872–881, Copyright 2021, with permission from Wiley. (Rosano et al., 2021).

In order to create the profiles, the HFA combined heart rate (HR<60, 60–70 or >70 beats per minute), blood pressure (BP, <90/60, >90/60 or >140/90 mmHg), atrial fibrillation (AF, yes/no), chronic kidney disease (CKD) and/or hyperkalemia (HK, serum potassium >5.0 mmol/L) which would add up to a total of 36 potential profiles when different categories of these parameters are combined. However, the HFA only based seven profiles on these categories, and added HR>60, potassium >5.5, estimated glomerular filtration rate (eGFR) >30 mL/min/1.73m2 or eGFR 30–60 mL/min/1.73m2 as new categories for their remaining four profiles, making a total of eleven patient profiles (Figure 1). This implies that 29 additional profiles based upon combinations of the fixed categories could be identified on top of the eleven HFA profiles for a total of 40 profiles. Therefore, we also analyzed these 29 additional profiles to explore the patients that could not be categorized into one of the HFA profiles.

The BP category <90/60 mmHg (hypotension) was defined as an SBP <90 mmHg and/or a DBP <60 mmHg while SBP had to be <140 mmHg. Hypertension was defined as an SBP ≥140 mmHg and/or DBP ≥90 mmHg. The BP category >90/60 mmHg (normotension) was defined as an SBP ≥90 mmHg but <140 mmHg and a DBP ≥60 mmHg but <90 mmHg. For heart rate, HR <60, HR 60–70, and HR >70 beats per minute were used as categories. CKD/HK was defined as an eGFR <60 30–60 mL/min/1.73 m^2^ and/or a serum potassium >5.0, while no CKD/HK was defined as an eGFR ≥60 and a serum potassium ≤5.0. Atrial fibrillation comprised all forms of AF (paroxysmal, permanent, persistent, unknown type).

## Results

**FIGURE 2:**
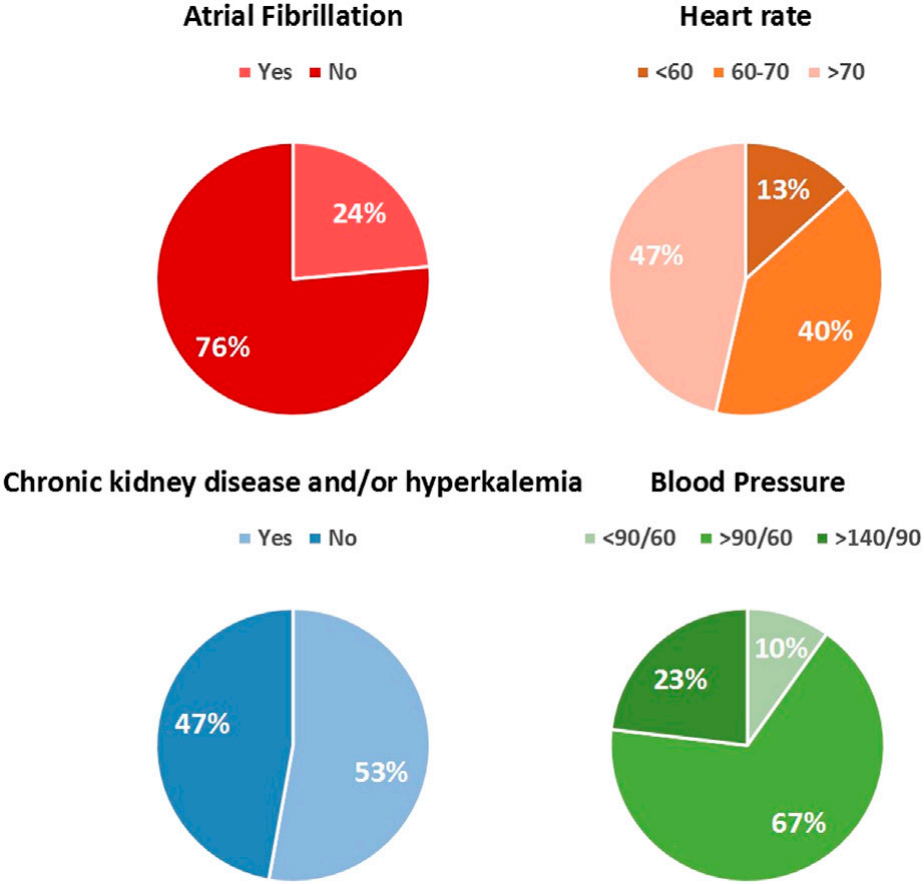
Distribution of the patient characteristics in a real-world chronic heart failure cohort.

A total of 4,455 patients with an LVEF ≤40% were analyzed. The distribution of the patient characteristics is shown in Figure 2. Importantly, 20% of all patients with CKD had an eGFR <30 mL/min/1.73 m^2^.

**FIGURE 3:**
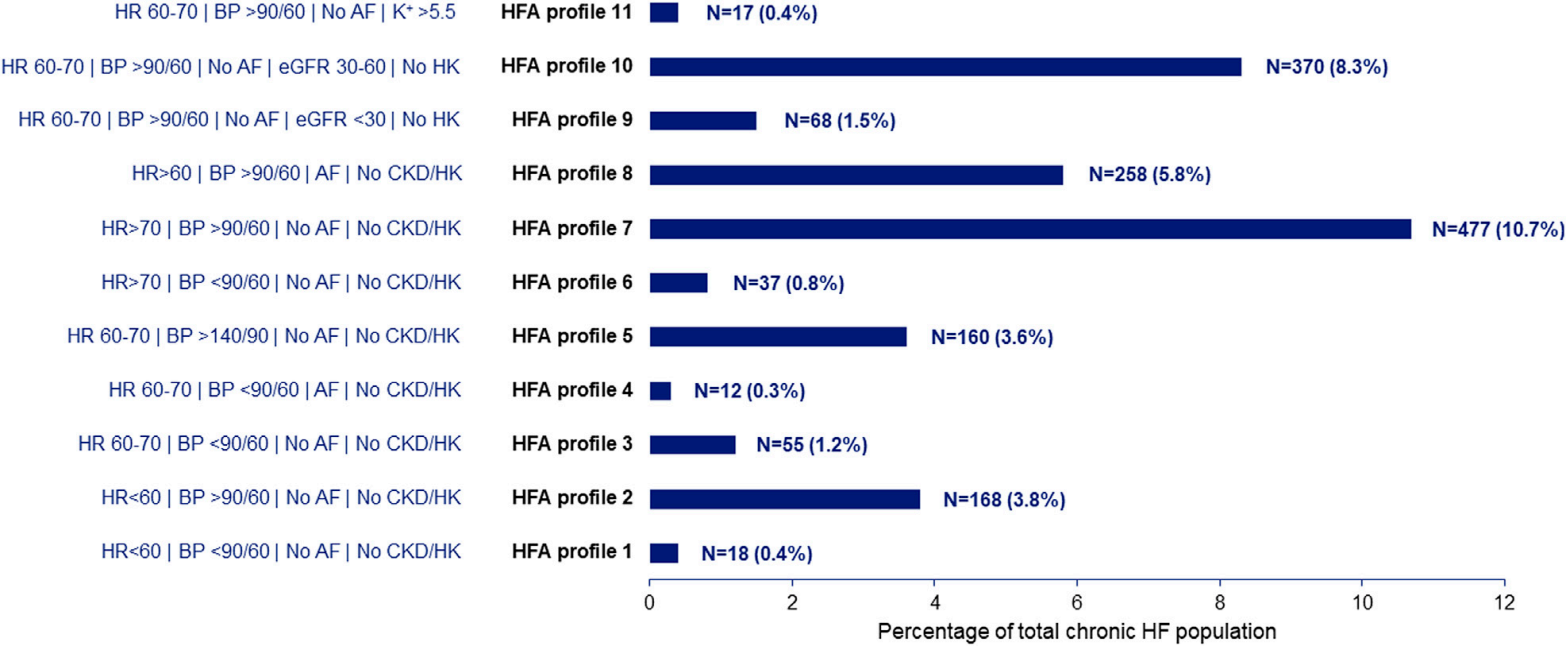
Prevalence of the patient profiles as proposed by the Heart Failure Association.

The eleven profiles postulated by the HFA comprised a total of 1,640 patients (36.8% of total population). Of these profiles, three seemed to be most relevant with a combined prevalence of 24.8%, all with a heart rate >60 beats per minute (bpm), BP >90/60 mmHg and no hyperkalemia. Detailed information about the prevalence of each profile is provided in Figure 3. Additional information on patient characteristics for each HFA profile is presented in Table 1. The most pronounced differences in patient characteristics between the HFA profiles were observed with respect to age, New York Heart Association class distribution and the presence of intraventricular conduction delay.

**TABLE 1:** Patient characteristics according to each HFA profile.

	Total (*N* = 4,455)	HFA profile 1	HFA profile 2	HFA profile 3	HFA profile 4	HFA profile 5	HFA profile 6
		HR <60	HR <60	HR 60–70	HR 60–70	HR 60–70	HR >70
		BP <90/60	BP >90/60	BP <90/60	BP <90/60	BP >140/90	BP <90/60
		No AF	No AF	No AF	AF	No AF	No AF
		No CKD/HK	No CKD/HK	No CKD/HK	No CKD/HK	No CKD/HK	No CKD/HK
		(N = 18)	(N = 168)	(N = 55)	(N = 12)	(N = 160)	(N = 37)
Age, median (IQR)	74 (16)	76 (20)	67 (17)	69 (25)	78 (16)	70 (16)	74 (15)
Male (%)	2,904 (65)	12 (67)	122 (73)	34 (62)	9 (75)	98 (61)	20 (54)
BMI, median (IQR)	27 (6)	23 (5)	26 (6)	26 (6)	24 (4)	27 (7)	23 (6)
eGFR, mL/min/1.73m2, median (IQR)	60 (38)	72 (19)	78 (21)	84 (30)	80 (33)	81 (21)	75 (25)
Serum potassium, median (IQR)	4.3 (0.6)	4.5 (0.4)	4.3 (0.6)	4.2 (0.7)	4.4 (0.5)	4.3 (0.6)	4.3 (0.6)
NYHA class (%)							
I	558 (13)	1 (6)	40 (24)	11 (20)	2 (17)	28 (18)	7 (19)
II	2,654 (60)	11 (61)	109 (65)	34 (62)	7 (58)	101 (63)	21 (57)
III	1,106 (25)	6 (33)	17 (10)	9 (16)	3 (25)	29 (18)	9 (24)
IV	92 (2)	0 (0)	1 (1)	1 (2)	0 (0)	2 (1)	0 (0)
LVEF, median (IQR)	30 (10)	27 (20)	30 (14)	30 (15)	28 (24)	30 (11)	23 (13)
Systolic BP, mmHg, median (IQR)	120 (26)	108 (21)	120 (19)	103 (16)	110 (25)	145 (18)	100 (22)
Diastolic BP, mmHg, median (IQR)	70 (18)	51 (5)	70 (12)	55 (7)	55 (7)	80 (12)	55 (8)
Heart rate, beats/min, median (IQR)	70 (18)	54 (4)	56 (6)	64 (5)	66 (9)	65 (5)	78 (11)
LBBB (%)	773 (17)	2 (11)	32 (19)	8 (15)	2 (17)	43 (27)	9 (24)
QRS≥130 ms (%)	1,564 (35)	5 (28)	51 (30)	21 (38)	5 (42)	60 (38)	12 (32)
NT-proBNP, pg/ml, median (IQR)	932 (2,384)	1,725 (10,702)	392 (944)	580 (2,811)	138*	455 (1,140)	1,387 (3,957)

	HFA profile 7	HFA profile 8	HFA profile 9	HFA profile 10	HFA profile 11
	HR >70	HR >60	HR 60–70	HR 60–70	HR 60–70
	BP >90/60	BP > 90/60	BP >90/60	BP >90/60	BP >90/60
	No AF	AF	No AF	No AF	No AF
	No CKD/HK	No CKD/HK	CKD (eGFR <30)No HK	CKD (eGFR 30–60)No HK	K+ >5.5
	(N = 477)	(N = 258)	(N = 68)	(N = 370)	(N = 17)
Age, median (IQR)	65 (18)	73 (12)	78 (11)	76 (13)	77 (12)
Male (%)	299 (63)	192 (74)	39 (57)	252 (68)	13 (77)
BMI, median (IQR)	27 (7)	27 (6)	26 (6)	27 (6)	27 (7)
eGFR, median (IQR)	82 (23)	80 (24)	25 (6)	47 (13)	32 (24)
Serum potassium, median (IQR)	4.2 (0.5)	4.2 (0.5)	4.4 (0.7)	4.4 (0.5)	5.7 (0.3)
NYHA class (%)					
I	61 (13)	27 (11)	1 (2)	28 (8)	8 (47)
II	311 (65)	155 (60)	30 (44)	219 (59)	4 (24)
III	97 (20)	71 (28)	35 (52)	111 (30)	4 (24)
IV	2 (0)	3 (1)	1 (2)	8 (2)	0 (0)
LVEF, median (IQR)	27 (13)	30 (12)	30 (10)	30 (10)	31 (13)
Systolic BP, mmHg, median (IQR)	120 (15)	120 (17)	120 (20)	120 (15)	117 (21)
Diastolic BP, mmHg, median (IQR)	70 (12)	70 (11)	70 (9)	70 (11)	66 (11)
Heart rate, beats/min, median (IQR)	79 (11)	80 (18)	64 (8)	65 (7)	64 (8)
LBBB (%)	81 (17)	47 (18)	9 (13)	57 (15)	4 (24)
QRS≥130 ms (%)	167 (35)	67 (26)	31 (46)	147 (40)	5 (29)
NT-proBNP, pg/ml, median (IQR)	479 (1,130)	907 (1,417)	1,353 (3,233)	778 (1,994)	695 (3,501)

HFA, heart failure association; BMI, body mass index; eGFR, estimated glomerular filtration rate; HR, heart rate; BP, blood pressure; AF, atrial fibrillation; CKD, chronic kidney disease, defined as an eGFR (estimated glomerular filtration rate) <60 mL/min/1.73m2; HK, hyperkalemia, defined as serum potassium >5.0 mmol/L; NYHA, new york heart association; LVEF, left ventricular ejection fraction; LBBB, left bundle branch block; NT-proBNP, N-terminal pro-brain natriuretic peptide; IQR, interquartile range.

The remaining 2,814 patients (63.2% of total population) could not be classified in the HFA profiles and were fitted into one of the 29 additional profiles. The prevalence of the additional profiles also varied strongly, but the largest four profiles together comprised nearly a third of the total population, and mostly consisted of patients with a BP >90/60 and CKD. Thirteen out of the 29 additional profiles had a prevalence lower than one percent of the population. Information about the additional profiles is presented in Supplementary Table S1.

Detailed information on medical therapy, including prescription rates and prescribed doses, for each HFA profile is shown in Table 2. As shown, there were between-profile differences in medical therapy which are discussed elaborately in the discussion section with comparisons to the recommendations as given by the HFA.

**TABLE 2:** Medical therapy use and dose according to each HFA profile.

	Total (*N* = 4,455)	HFA profile 1	HFA profile 2	HFA profile 3	HFA profile 4	HFA profile 5
		HR <60	HR <60	HR 60–70	HR 60–70	HR 60–70
		BP <90/60	BP >90/60	BP <90/60	BP <90/60	BP >140/90
		No AF	No AF	No AF	AF	No AF
		No CKD/HK	No CKD/HK	No CKD/HK	No CKD/HK	No CKD/HK
		(N = 18)	(N = 168)	(N = 55)	(N = 12)	(N = 160)
**Beta blocker** ^a^						
Use	3,548 (80.1)	15 (83.3)	137 (82.0)	48 (87.3)	9 (75.0)	133 (83.1)
Daily dose						
<50%	1,636 (46.3)	10 (66.7)	68 (49.6)	22 (45.8)	5 (55.6)	61 (46.6)
50%–99%	1,250 (35.4)	5 (33.3)	50 (36.5)	18 (37.5)	3 (33.3)	42 (32.1)
≥100%	649 (18.4)	0 (0)	19 (13.9)	8 (16.7)	1 (11.1)	28 (21.4)
**MRA** ^a^						
Use	2,527 (57.0)	11 (61.1)	111 (66.5)	37 (67.3)	9 (75.0)	73 (45.6)
Daily dose						
<50%	849 (33.8)	6 (54.5)	31 (28.2)	12 (32.4)	4 (44.4)	29 (40.3)
50%–99%	1,416 (56.3)	5 (45.5)	74 (67.3)	22 (59.5)	5 (55.6)	38 (52.8)
≥100%	249 (9.9)	0 (0)	5 (4.5)	3 (8.1)	0 (0)	5 (6.9)
**RAS inhibitor** ^a^						
Use	3,657 (82.5)	13 (72.2)	153 (91.6)	45 (81.8)	8 (66.7)	140 (87.5)
Daily dose						
<50%	900 (24.7)	4 (33.3)	35 (22.9)	15 (33.3)	1 (12.5)	20 (14.3)
50%–99%	1,136 (31.2)	5 (41.7)	50 (32.7)	15 (33.3)	2 (25.0)	27 (19.3)
≥100%	1,605 (44.1)	3 (25.0)	68 (44.4)	15 (33.3)	5 (62.5)	93 (66.4)
**Diuretics** ^a^						
Use	3,752 (84.7)	15 (83.3)	121 (72.5)	51 (92.7)	10 (83.3)	114 (71.7)

	HFA profile 6	HFA profile 7	HFA profile 8	HFA profile 9	HFA profile 10	HFA profile 11
	HR >70	HR >70	HR >60	HR 60–70	HR 60–70	HR 60–70
	BP <90/60	BP >90/60	BP > 90/60	BP >90/60	BP >90/60	BP >90/60
	No AF	No AF	AF	No AF	No AF	No AF
	No CKD/HK	No CKD/HK	No CKD/HK	CKD (eGFR <30)No HK	CKD (eGFR 30–60)No HK	K^+^ >5.5
	(N = 37)	(N = 477)	(N = 258)	(N = 68)	(N = 370)	(N = 17)
**Beta blocker** ^a^						
Use	29 (78.4)	377 (79.4)	209 (81.0)	55 (83.3)	300 (81.1)	16 (94.1)
Daily dose^a^						
<50%	17 (60.7)	181 (48.1)	74 (35.6)	28 (51.9)	159 (53.2)	7 (43.8)
50%–99%	7 (25.0)	121 (32.2)	85 (40.9)	16 (29.6)	92 (30.8)	6 (37.5)
≥100%	4 (14.3)	74 (19.7)	49 (23.6)	10 (18.5)	48 (16.1)	3 (18.8)
**MRA** ^a^						
Use	20 (54.1)	284 (59.8)	150 (58.1)	43 (65.2)	233 (63.0)	5 (29.4)
Daily dose^a^						
<50%	6 (30.0)	97 (34.3)	44 (29.9)	11 (25.6)	77 (33.2)	5 (100.0)
50%–99%	10 (50.0)	154 (54.4)	82 (55.8)	24 (55.8)	139 (59.9)	0 (0)
≥100%	4 (20.0)	32 (11.3)	21 (14.3)	8 (18.6)	16 (6.9)	0 (0)
**RAS inhibitor** ^a^						
Use	28 (75.7)	414 (87.2)	202 (78.3)	50 (75.8)	318 (85.9)	14 (82.4)
Daily dose^a^						
<50%	11 (39.3)	103 (25.1)	60 (29.6)	17 (34.0)	84 (26.6)	4 (28.6)
50%–99%	7 (25.0)	144 (35.0)	76 (37.4)	16 (32.0)	96 (30.4)	3 (21.4)
≥100%	10 (35.7)	164 (39.9)	67 (33.0)	17 (34.0)	136 (43.0)	7 (50.0)
**Diuretics**						
Use	32 (86.5)	381 (80.2)	215 (83.3)	63 (95.5)	340 (91.9)	14 (82.4)

^a^ The numbers presented have taken into account missing data on use and the prescribed dose. Daily dose is displayed as percentage of the guideline-recommended target dose. HFA, heart failure association; BMI, body mass index; eGFR, estimated glomerular filtration rate; HR, heart rate; BP, blood pressure; AF, atrial fibrillation; CKD, chronic kidney disease, defined as an eGFR (estimated glomerular filtration rate) <60 mL/min/1.73m2; HK, hyperkalemia, defined as serum potassium >5.0 mmol/L; MRA, mineralocorticoid receptor antagonist; RAS, renin-angiotensin system.

## Discussion

According to the latest HF guidelines, clinicians have four cornerstone drugs with a Class 1 recommendation for the treatment of patients with HFrEF. (McDonagh et al., 2021). The general consensus on the best strategy to initiate and up titrate the cornerstone drugs is currently being reconsidered as rapid drug sequencing seems more desirable than the conventional approach. (Packer and McMurray, 2021). The current guidelines also emphasize initiation of all four HF drugs as early as possible. (McDonagh et al., 2021). A thorough analysis of several landmark HF trials by Kondo et al. has shown that a considerable proportion of the patients who were enrolled in the clinical trials did not actually reach the target dose as recommended in the guidelines. (Kondo et al., 2022). Nonetheless, the lower doses were also shown to be effective shortly after drug initiation, which supports initiating all four drug classes as rapidly as possible at low dose rather than fully up-titrating a drug class before adding a new one. A recent analysis of the Swedish Heart Failure Registry showed that the use of two drug classes at 50%–99% of the target dose was associated with lower risk of HF events than a single drug class at full target dose. (D'Amario et al., 2022). Furthermore, the four HF drugs combined have been proven superior over other combinations and single drug-use in an extensive network meta-analysis by Tromp et al. (Tromp et al., 2022)

While the increasing body of evidence suggests initiating all four drug classes as rapidly as possible, there are several possible strategies to achieve this. Beldhuis et al. suggest starting the drug classes in the order of SGLT2i, MRA, ARNI, and then betablocker as fast as possible. (Beldhuis et al., 2022). Packer et al. instead advocate starting with a betablocker and SGLT2i, followed by ARNI and MRA in whatever order. (Packer and McMurray, 2021). Greene et al. postulated that initiating all four drug classes simultaneously or rapidly sequential at low doses and titration to target dose afterwards would provide the best results. (Greene et al., 2021). In contrast to these viewpoints, the HFA presented patient profiling as alternative “tailored medicine” strategy to achieve GDMT in a personalized way rather than by fixed sequencing approaches. (Rosano et al., 2021). Considering the large heterogeneity in phenotypes, HF patients may require different strategies for optimal drug implementation. This warrants a personalized approach and the profiles as postulated by the HFA may assist in this process by tailoring the sequence of titration of particular drugs to the patient’s phenotype to achieve the best treatment for individual patients that may not tolerate all four drugs at the recommended doses. (Rosano et al., 2021). This personalized approach allows clinicians to initiate a regimen as close as possible to GDMT in each patient, and may thereby improve prognosis. The HFA has provided recommendations for several different phenotypes that may be relevant for multiple patient profiles.

### The phenotype-specific recommendations and drug therapy in our chronic heart failure cohort

1. Patients with low blood pressure and high heart rate: This hemodynamic phenotype includes HFA profile 6 (0.8% prevalence). The HFA recommends modification of GDMT in case of symptomatic hypotension, which is in line with the practical guidance of the latest guidelines. For this profile, the HFA recommends that, despite low systolic blood pressure, betablockers should be up-titrated to the target dose or maximum tolerated dose. Furthermore, they state that MRAs have a minimal effect on blood pressure, and rarely need to be discontinued. Our results show that there is still much room for implementation and up-titration of betablockers and, mainly, of MRAs (prescribed in 54.1% of the patients of whom only 20% received the guideline-recommended dose), especially considering the fact that the patients in this profile had a normal kidney function.
2. Patients with low blood pressure and low heart rate: This phenotype corresponds with HFA profile 1 (0.4% prevalence), and according to the recommendations, withdrawal of MRAs is not necessary, while reduction of betablocker dose may be necessary in case of a heart rate <50 bpm or symptomatic bradycardia. Our analysis showed that 83.3% of the patients in this profile were treated with a betablocker, but only 57% received an MRA which indicates possibilities for further treatment optimization.
3. Patients with normal blood pressure and low heart rate: This phenotype refers to HFA profile 2, which had a prevalence of 3.8% in our study. The HFA recommends betablockers and/or ivabradine to be down-titrated in case of symptomatic bradycardia or heart rate <50 bpm. Eighty-two percent of the patients in this profile were treated with a betablocker, of whom 13.9% received the guideline-recommended dose. As blood pressure and kidney function were normal in this profile, there is the possibility of up-titration of ACEi/ARB and MRA.
4. Patients with normal blood pressure and high heart rate: This phenotype corresponds with HFA profile 7, which was one of the most relevant profiles with a prevalence of nearly 11%. The HFA recommends treatment with target doses of betablockers, and up-titration of ACEi/ARB or ARNI to target dose as well. Our results show that 79.4% of the patients in this profile were treated with a betablocker, of whom only 19.7% received the guideline-recommended dose. Considering the high heart rate (>70 bpm) and absence of atrial fibrillation, it is striking that such a small proportion of patients were treated with guideline-recommended betablocker therapy. Furthermore, whilst 87.2% were treated with ACEi/ARB, only 39.9% received the target dose, which indicates room for further improvement, especially in light of absence of chronic kidney disease.
5. Patients with atrial fibrillation and normal blood pressure: This phenotype is captured in HFA profile eight and was quite common with a prevalence of 5.8%. Atrial fibrillation is frequently seen in patients with chronic HF, which was also shown in our cohort with a prevalence of 24% (Figure 2). The HFA states that the ideal resting heart rate is not yet clear, but that it may be between 60–80 bpm. While clear evidence for beneficial effects of betablockers in HF patients with AF is lacking, it is believed that ventricular rates <70 bpm are associated with worse outcome. Interestingly, 81% of the patients in this profile were treated with a betablocker, and the average heart rate was 80 bpm, so there is still room for optimization according to the HFA’s recommendations.
6. Patients with atrial fibrillation and low blood pressure: This phenotype was described as HFA profile 4, which was very uncommon in our population (prevalence of 0.3%). The average heart rate was 66 bpm, whereas a heart rate >70 bpm should be aimed for according to the recommendations. The HFA recommends betablockers to be reduced or discontinued if necessary. Our results indicated that 75% used a betablocker of whom 11.1% at the full guideline-recommended dose, and with an average heart rate of 66 bpm, it appears that down-titration of betablockers in this profile could be beneficial. The expected increase in blood pressure could enable initiation and up-titration of ACEi/ARB as only 66.7% received these, of whom 62.5% at the recommended dose. Strikingly, 75% used an MRA, but no patient received the recommended dose.
7. Patients with chronic kidney disease (CKD): As shown in our cohort, CKD is frequently encountered in daily clinical practice, and it may inhibit GDMT implementation. HFA profiles 9 and 10 consisted of patients with CKD (including patients with an eGFR<30 and eGFR 30–60), and together accounted for 9.8% of our cohort. The HFA stated that all foundational HF drugs can be given down to an eGFR of 30. However, our analysis showed that in HFA profile 10 (eGFR 30–60), only 81.1% used a betablocker, 63% an MRA and 85.9% an ACEi/ARB, and mainly at lower than recommended doses. Therefore, there appears to be ample room for drug optimization in this particular profile. Interestingly, diuretics were often prescribed in these profiles (95.% and 91.9% for HFA profiles 9 and 10, respectively).
8. Pre-discharge patient: Unfortunately, the current registry only included patients in outpatient setting, so information for this subgroup was unavailable.
9. Patients with hypertension despite guideline-directed medical therapy: As shown in Figure 2, 23% of our cohort suffered from hypertension (defined as either systolic blood pressure >140, diastolic blood pressure >90, or both). The HFA recommends optimal doses of GDMT in this phenotype. Hypertension is captured in HFA profile 5. Strikingly, despite high blood pressure, normal heart rate and absence of AF, CKD and HK, only 83.1% used a betablocker, of whom 21.4% at the optimal dose, 45.6% used an MRA, of whom 6.9% at full recommended dose, and 87.5% used an ACEi/ARB, but only 66.4% at the recommended dose. Therefore, there appears to be plenty room for further optimization of GDMT in this particular profile.

As discussed above, the HFA provided treatment recommendations for several phenotypes that may encompass multiple patient profiles. In this study, we showed that the HFA profiles comprise nearly forty percent of the population. The added value of patient profiling is to provide more specific recommendations on top of general guideline recommendations for patients that may require a more personalized approach. We have also identified additional profiles that together accounted for 63% of our HF population. In their position paper, the HFA clearly stated that patients may not always be fitted into one particular profile based upon these parameters, and that certain profiles may need to be combined and compared for personalized advice. (Rosano et al., 2021). Considering that the HFA has provided recommendations for broader phenotypes rather than for each individual patient profile, we believe that the recommendations as provided by the HFA can to an extent be applied to the additional profiles as well. For example, the hypertension and CKD phenotypes are broadly defined and comprise multiple patient profiles, including a large proportion of the additional patient profiles. Moreover, a considerable proportion of the patients who were fitted into the additional profiles did not have characteristics that required deviation from the general recommendations in the ESC HF Guidelines (for example, Additional profile 1). (McDonagh et al., 2021). Finally, it needs to be mentioned that enlarging the number of possible profiles may have the unwanted effect of complicating the application of this approach and may therefore reduce the usefulness of phenotypic profiling.

### Future perspectives

In short, our analysis showed that application of patient profiling with corresponding phenotype-specific treatment recommendations may be beneficial for implementation of GDMT in real-world clinical practice. However, it is important to mention that, although we were able to study treatment by profile, the data presented are cross-sectional, and it was unknown whether side effects or tolerability may have influenced prescription of HF drugs. Our study may serve as an important platform to expand our knowledge of patient profiling, and may contribute to its use in clinical practice. Future studies investigating the barriers to guideline implementation, and the optimal strategy for drug sequencing based on phenotypic profile are needed to improve our understanding of the titration process in real-world setting and may improve implementation of guideline-recommended pharmacological HF therapy.

## Conclusion

Nearly forty percent of a real-world chronic HF population was classified in the eleven patient profiles as suggested by the HFA. Phenotype-specific treatment recommendations are clinically relevant and important to further improve guideline implementation.

## Data Availability

The original contributions presented in the study are included in the article/Supplementary Materials, further inquiries can be directed to the corresponding author.
