# Supplementary material for: Phenotypic patient profiling for improved implementation of guideline-directed medical therapy: An exploratory analysis in a large real-world chronic heart failure cohort
Source: Front Pharmacol. 2023 Mar 9;14:1081579. doi: 10.3389/fphar.2023.1081579 (PMC10033992; doi:10.3389/fphar.2023.1081579)
Supplement: Supplementary file 1 [file Table1.DOCX]

**Supplementary Table 1. Prevalence of the additional patient profiles**

| **Additional profile 1**  HR 60-70  BP >90/60  No AF  No CKD/HK  **N = 509 (11.4% of total)** | **Additional profile 2**  HR >70  BP >90/60  No AF  CKD/HK  **N = 483 (10.8% of total)** | **Additional profile 3**  HR >70  BP >90/60  AF  CKD/HK  **N = 248 (5.6% of total)** | **Additional profile 4**  HR 60-70  BP >140/90  No AF  CKD/HK  **N = 181 (4.1% of total)** | **Additional profile 5**  HR >70  BP >140/90  No AF  CKD/HK  **N = 169 (3.8% of total)** | **Additional profile 6**  HR >70  BP >140/90  No AF  No CKD/HK  **N = 167 (3.7% of total)** |
| --- | --- | --- | --- | --- | --- |
| **Additional profile 7**  HR <60  BP >90/60  No AF  CKD/HK  **N = 130 (2.9% of total)** | **Additional profile 8**  HR 60-70  BP >90/60  AF  CKD/HK  **N = 108 (2.4% of total)** | **Additional profile 9**  HR >70  BP <90/60  No AF  CKD/HK  **N = 95 (2.1% of total)** | **Additional profile 10**  HR 60-70  BP <90/60  No AF  CKD/HK  **N = 94 (2.1% of total)** | **Additional profile 11**  HR >70  BP >140/90  AF  CKD/HK  **N = 81 (1.8% of total)** | **Additional profile 12**  HR >70  BP>140/90  AF  No CKD/HK  **N = 65 (1.5% of total)** |
| **Additional profile 13**  HR <60  BP >140/90  No AF  No CKD/HK  **N = 63 (1.4% of total)** | **Additional profile 14**  HR 60-70  BP >90/60  No AF  CKD/HK  **N = 55 (1.2% of total)** | **Additional profile 15**  HR <60  BP >140/90  No AF  CKD/HK  **N = 54 (1.2% of total)** | **Additional profile 16**  HR <60  BP >90/60  AF  CKD/HK  **N = 43 (1.0% of total)** | **Additional profile 17**  HR <60  BP <90/60  No AF  CKD/HK  **N = 37 (0.8% of total)** | **Additional profile 18**  HR >70  BP <90/60  AF  CKD/HK  **N = 38 (0.9% of total)** |
| **Additional profile 19**  HR 60-70  BP >140/90  AF  CKD/HK  **N = 36 (0.8% of total)** | **Additional profile 20**  HR <60  BP >90/60  AF  No CKD/HK  **N = 33 (0.7% of total)** | **Additional profile 21**  HR 60-70  BP>140/90  AF  No CKD/HK  **N = 26 (0.6% of total)** | **Additional profile 22**  HR 60-70  BP <90/60  AF  CKD/HK  **N = 21 (0.5% of total)** | **Additional profile 23**  HR 60-70  BP >90/60  AF  No CKD/HK  **N = 19 (0.4% of total)** | **Additional profile 24**  HR <60  BP>140/90  AF  No CKD/HK  **N = 15 (0.3% of total)** |
| **Additional profile 25**  HR <60  BP >140/90  AF  CKD/HK  **N = 14 (0.3% of total)** | **Additional profile 26**  HR >70  BP <90/60  AF  No CKD/HK  **N = 14 (0.3% of total)** | **Additional profile 27**  HR <60  BP <90/60  AF  CKD/HK  **N = 12 (0.3% of total)** | **Additional profile 28**  HR <60  BP <90/60  AF  No CKD/HK  **N = 5 (0.1% of total)** | **Additional profile 29**  HR >70  BP >90/60  AF  No CKD/HK  **N = 0 (0% of total)** |  |

HFA, Heart Failure Association; HR, heart rate; BP, blood pressure; AF, atrial fibrillation; CKD, chronic kidney disease, defined as an eGFR (estimated glomerular filtration rate) <60 mL/min/1.73m^2^; HK, hyperkalemia, defined as serum potassium >5.0 mmol/L
